# MicroRNAs in Rice Innate Immunity

**DOI:** 10.1186/s12284-016-0078-5

**Published:** 2016-02-20

**Authors:** Patricia Baldrich, Blanca San Segundo

**Affiliations:** Centre for Research in Agricultural Genomics (CRAG) CSIC-IRTA-UAB-UB, Carrer de la Vall Moronta, Edifici CRAG, Campus UAB, Bellaterra (Cerdanyola del Vallés), Barcelona, 08193 Spain

**Keywords:** Disease resistance, *Magnaporthe oryzae*, MicroRNA, Pathogen, Rice improvement

## Abstract

MicroRNAs (miRNAs) are short regulatory non-coding RNAs that guide gene silencing in most eukaryotes. They regulate gene expression by triggering sequence-specific cleavage or translational repression of target transcripts. Plant miRNAs are known to play important roles in a wide range of developmental processes. Increasing evidence also supports that the modulation of miRNA levels plays an important role in reprogramming plant responses to abiotic stress (drought, cold, salinity and nutrient deficiency) and biotic stress (antibacterial resistance). Most of these studies were carried out in the model plant *Arabidopsis thaliana*. During the last years, the adoption of high-throughput sequencing technologies has significantly contributed to uncover multiple miRNAs while allowing miRNA profiling in plants. However, although a plethora of rice miRNAs have been shown to be regulated by pathogen infection, the biological function remains largely unknown for most of them. In this review, we summarize our current understanding on the contribution of miRNAs to rice immunity and discuss their potential applications in rice biotechnology. A better understanding of the miRNA species controlling rice immunity may lead to practical biotechnological applications leading to the development of appropriate strategies for rice protection.

## Introduction

Small RNAs (sRNAs) are short non-coding RNAs that guide gene silencing in most eukaryotes (Baulcombe 2004; Vaucheret 2006). Plants have two main classes of sRNAs, microRNAs (miRNAs) and small interfering RNAs (siRNAs), which are distinguished by their mode of biogenesis and mechanisms of action. Plant miRNAs are transcribed from *MIR* genes by RNA polymerase II as long single-stranded RNA precursors with unique stem-loop structures, the primary-miRNAs (pri-miRNAs) which are then processed by RNAse III DICER-LIKE proteins (DCL) to give rise to double stranded miRNA duplexes (miRNA/miRNA*, also named miRNA-5p/miRNA-3p duplexes) (Kurihara and Watanabe 2004). The two strands of the miRNA/miRNA* duplex are methylated at their 3’ end and transported from the nucleus into the cytoplasm, where the functional miRNA is loaded into the RNA-induced silencing complex (RISC) that contains the ARGONAUTE1 (AGO1) protein as the core component. MiRNAs guide post-transcriptional gene silencing through sequence-specific cleavage or translational repression of target mRNAs (Llave et al. 2002; Brodersen et al. 2008). In addition to miRNAs, plants produce different types of endogenous siRNAs which can be further classified into at least two major classes, phased siRNAs (phasiRNAs) and heterochromatic siRNAs (hc-siRNAs) (Arikit et al. 2013; Axtell 2013). SiRNAs differ from miRNAs in that they arise from double-stranded RNAs that originate through the action of RNA-dependent RNA polymerases (RDRs).

The production of each class of small RNAs has its own requirements for RDR and DCL proteins. DCL1 is mainly involved in the generation of miRNAs, although alternative pathways for miRNA biogenesis involving DCL3 or DCL4 have also been described (Rajagopalan et al. 2006; Vazquez et al. 2008). DCL3 is responsible for the processing of RDR2-generated double stranded RNAs and gives rise to hc-siRNAs (Xie et al. 2004). As for AGO proteins, AGO1 primarily binds miRNAs whereas AGO4 binds hc-siRNAs (Zilberman et al. 2003; Baumberger and Baulcombe 2008).

Numerous studies in different plant species have demonstrated the crucial role of miRNAs in a wide range of plant developmental processes, such as organ polarity and morphogenesis, flowering, shoot and root development, and hormone signalling, among others (Palatnik et al. 2003; Mallory et al. 2004; Chen 2009; Rubio-Somoza and Weigel 2011). There are also reports indicating that miRNAs respond to different types of abiotic and biotic stress (Chiou et al. 2006; Navarro et al. 2006; Sunkar and Zhu 2004; Jeong and Green 2013; Staiger et al. 2013; Yang and Huang 2014). Most research on plant miRNAs has been conducted in the model dicotyledonous plant *Arabidopsis thaliana*.

Rice (*Oryza sativa*) is the most widely consumed staple food for a large part of the world’s human population, providing a major portion of calories in human diet. One of the major factors limiting rice production is the occurrence of diseases caused by various fungal, bacterial and viral pathogens. Rice blast (*Magnaporthe oryzae*) and sheath blight (*Rhizoctonia solani*) are the two most devastating fungal diseases of rice (*Oryza sativa*). Bakanae (“foolish seedling” in Japanese) caused by the fungus *Fusarium* spp *(Gibberella fujikuroi* species complex) is also common rice disease. Rice yields can be also severely compromised by the bacterial pathogens *Xanthomonas oryzae* pv. *oryzae* and *X. oryzae* pv *oryzicola* (bacterial blight and bacterial leaf streak, respectively). More recently, bacterial foot rot (*Dickeya zeae*, previously known as *Erwinia chrysanthemi* pv. *zeae*) and bacterial panicle blight of rice (*Burkholderia glumae*) have emerged as important pathogens affecting global rice production. Rice virus diseases are generally restricted to specific rice-growing areas, *e.g*., Rice Hoja Blanca Virus (RHBH) in South-America, and Rice Yellow Mottle Virus (RYMV) in Africa. Rice stripe virus, Rice dwarf virus, and Rice tungro viruses (RSV, RDV, RTBV, RTSV) are found in Asia. A practical means of achieving greater yields in rice is to develop strategies to minimize losses due to diseases.

In recent years, a significant progress has been made in the identification of miRNAs from rice, a monocotyledonous species that has been adopted as the model cereal for functional genomics. The relevance of distinct rice miRNAs in controlling traits of agronomic importance, such as tiller growth, early flowering, panicle and grain production, is well demonstrated (Miura et al. 2010; Wang et al. 2012; Zhang et al. 2013). In this review, we focus on the function of miRNAs in plant immunity, with special emphasis on rice miRNAs. Additionally, we describe the potential of miRNA-based technologies to improve disease resistance in rice, including transgenic and genome editing methods and the use of artificial miRNA or target mimicry techniques.

## Review

### MicroRNAs and other small RNAs in plant innate immunity

Plants have evolved a multilayered innate immune system to defend themselves against pathogens. The first line of defence occurs through recognition of conserved Pathogen Associated Molecular Patterns (PAMPs) by host Pattern-Recognition Receptors (PRR). Sensing PAMPs triggers a general defence response referred to as PAMP-triggered immunity (PTI), which operates against most pathogens. Among others, PTI components include deposition of callose, production of reactive oxygen species (ROS), activation of protein phosphorylation/dephosphorylation processes and accumulation of Pathogenesis-related proteins (PRs) (Jones and Dangl 2006; Boller and He 2009). To counteract this innate defence, pathogens produce effectors that suppress PTI. In turn, many plants have evolved another layer of immunity triggered by Resistance (R) proteins, the so-called Effector-Trigered Immunity (ETI). This type of immunity relies on the specific recognition of microbial effectors (or host proteins modified by effectors) by proteins encoded by *R* genes that trigger a rapid and effective host defence response. The essential role of the phytohormones salicylic acid (SA), ethylene (ET), jasmonic acid (JA) and abscisic acid (ABA) in resistance to pathogens is well established in plants (Denancé et al. 2013).

In rice, resistance to bacterial and fungal pathogens is conferred by both resistance genes (ETI) and basal resistance (PTI) (Liu et al. 2014). Concerning blast disease resistance, a broad array of blast resistance (*R*) genes have been described and are being effectively used in breeding programs to increase resistance to the rice blast fungus. However, rice improvement for durable resistance to blast based on *R* genes is difficult as most of the resistance genes break down in a few years because of the race specificity and the rapid change in pathogenicity of the blast fungus.

**Fig. 1:**
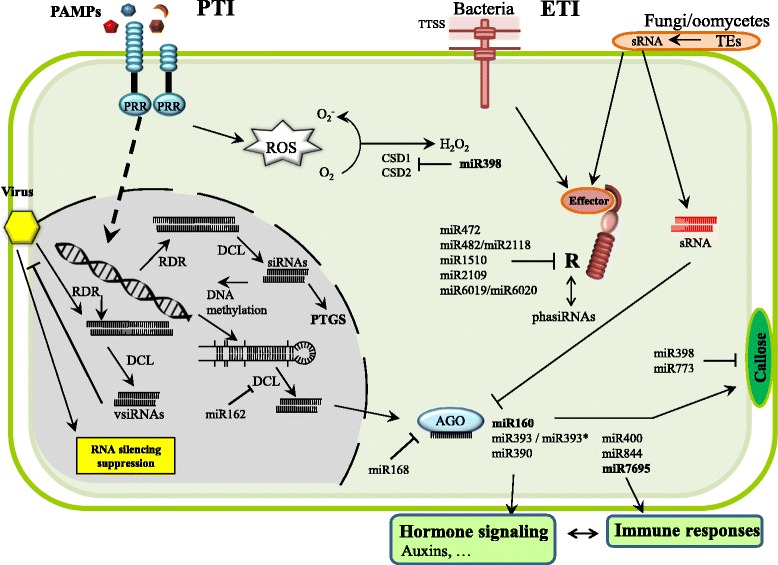
Functionally characterized miRNAs in relation to disease resistance. Most of the indicated miRNAs have been characterized in Arabidopsis plants infected with *Pseudomonas syringae*. Dicer-like proteins (DCLs) process double-stranded RNAs or single-stranded RNAs with hairpin structures, giving rise to mature sRNAs which are loaded into Argonaute (AGO)-containing RNA induced silencing complexes (RISCs) to induce gene silencing of their complementary targets. The RNA silencing-based antiviral defence involves the production of virus-derived sRNAs (vsiRNAs) by DCL activities that drive degradation of viral RNA in a sequence-specific manner. In addition to post-transcriptional gene silencing (PTGS)-based regulation, a role for RNA-directed DNA methylation (RdDM) in plant disease has emerged. Two miRNAs, miR162 and miR168, are involved in the regulation of the miRNA pathway itself by guiding cleavage of DCL1 and AGO1 mRNAs, respectively. In rice, the involvement of miR160, miR398 and miR7695 (in bold) in PTI responses is documented (Campo et al. 2013; Li et al. 2013). miR160 targets Auxin Response Factors (ARFs) whereas miR398 targets Cu/Zn superoxide dismutase genes. MiR7695 targets an alternatively spliced transcript of the *Natural resistance-associated macrophage protein 6* (*Nramp6*) gene from rice. In the case of miR393, the two sRNAs of the duplex are functional during pathogen infection (miR393 targets *TIR1*, whereas miR393* regulates MEM12, a Golgi-localised SNARE protein that modulates exocytosis of antimicrobial PR1 proteins in Arabidopsis (Zhang et al. 2011). ETI, Effector-Triggered Immunity; PAMPs, Pathogen-associated molecular pattern; PRR, Pattern recognition receptor; PTI, PAMP-Triggered Immunity; TTSS, Type three secretion system; RDR, RNA-dependent RNA polymerase; sRNA, small RNA; TEs, Transposable elements; ROS, Reactive oxygen species; CDS, Copper/Zinc superoxide dismutase

PTI and ETI responses to bacterial and fungal pathogens have been historically considered as protein-based defence mechanisms that are regulated at the transcriptional level, largely independent from the RNA-based mechanisms that typically operate in antiviral defence. Increasing evidence supports that plants also use post-transcriptional regulation of immune responses triggered by fungal and bacterial pathogens (Navarro et al. 2006; Katiyar-Agarwal and Jin 2010; Pumplin and Voinnet 2013; Seo et al. 2013; Staiger et al. 2013). The involvement of miRNAs in PTI responses and pathogen resistance was first demonstrated in Arabidopsis where perception of flg22, a peptide derived from the general elicitor flagellin, causes an increase in miR393 accumulation which in turn negatively regulates transcripts for F-box auxin receptors. MiR393-mediated repression of auxin signalling results in bacterial resistance (Navarro et al. 2006) (Fig. 1). This study clearly established a link between miRNA functioning, hormone signalling and immunity in Arabidopsis plants. Thus, in addition to controlling host developmental processes, a pathogen-regulated accumulation of miRNAs involved in hormone signalling might contribute to regulation of defence responses directly or indirectly via cross-talk between defence-related hormones.

Moreover, a miRNA-guided regulation of *R* gene expression during ETI responses has been demonstrated (Fig. 1). Several miRNAs have been shown to guide cleavage of *R* genes in *Solanaceae* and *Leguminosae* species as well as in Arabidopsis (Padmanabhan et al. 2009; Jagadeeswaran et al. 2009; Shivaprasad et al. 2012; Boccara et al. 2014). This is the case of miR482/miR2118 and miR6019/miR6020, among others (Fig. 1, and discussed below). Thus, it appears that miRNAs might function during PTI and/or ETI responses. *A priori*, pathogen-induced miRNAs might target negative regulators of the plant response to pathogen infection whereas pathogen-repressed miRNAs might target positive regulators.

In addition to miRNAs themselves, components of the miRNA biogenesis and functioning pathway are known to play a role in disease resistance (Seo et al. 2013). For instance, the Arabidopsis *dcl1-9* mutant (defective in miRNA biogenesis) and *ago* mutants (defective in miRNA activity) are impaired in PTI and display enhanced susceptibility to bacterial infection, whereas the Arabidopsis *dcl1-7* mutant is more susceptible to infection by the fungal pathogen *Botrytis cinerea* (Weiberg et al. 2014) Also in Arabidopsis, the *rdr6* mutant is more susceptible to bacterial and fungal infection (Katiyar-Agarwal and Jin 2010; Ellendorff et al. 2009). The Arabidopsis AGO1 is required for *Verticillium* pathogenicity (Ellendorff et al. 2009), whereas AGO2 is involved in pathogen immunity by binding miR393* in modulating the exocytosis of Pathogenesis-Related 1 (PR1) (Zhang et al. 2011).

The function of hc-siRNA-mediated transcriptional regulation in plant immunity has also come to light as pathogen infection or treatment with fungal elicitors induce alterations in the accumulation of certain hc-siRNAs, this type of sRNAs triggering RNA-directed DNA methylation (RdDM) (Dowen et al. 2012; Baldrich et al. 2015) (Fig. 1). It has been proposed that DNA methylation is part of the Arabidopsis immune response during antibacterial resistance by priming transcriptional activation of some defence genes (Dowen et al. 2012; Yu et al. 2013). The contribution of AGO4, a component of the RdDM pathway, in plant immunity is also documented (Franco-Zorrilla et al. 2007; López et al. 2011).

RNA silencing functions as a natural antiviral defence mechanism. It involves the production of virus-derived small interfering RNAs that drive degradation of viral RNAs in a sequence-specific manner. Antiviral silencing starts with the DCL-dependent production of viral siRNAs (vsiRNAs) (Hamilton and Baulcombe 1999; Parent et al. 2015) (Fig. 1). Similar to miRNAs, viral-derived siRNAs are incorporated into AGO1-containing RISC leading to inactivation of viral RNAs by cleavage. A host RDR then recognizes cleaved viral transcripts and secondary vsiRNAs are produced. This amplification mechanism can boost vsiRNA production, and systemically protect the plant against the same virus at distant sites. The involvement of AGO proteins in antiviral defence is well documented, AGO1 and AGO2 acting in a synergic manner in viral RNA degradation and AGO4 in viral DNA methylation (Mallory et al. 2004; Várallyay et al. 2010; Harvey et al. 2011; Scholthof et al. 2011; Wang et al. 2012). In turn, viruses activate counter-defence mechanisms to circumvent host antiviral RNA silencing mechanisms by expressing RNA-silencing suppressors or adopting silencing-resistant RNA structures. Since the first identified viral RNA silencing suppressor (VSR) (Ding et al. 1996), several VSRs have been characterized in rice viral pathogens (i.e. the Rice stripe virus NS3, Rice Yellow Mottle Virus P1, Rice Dwarf Virus PNS10, or Rice Stripe Virus P2 suppressors) (Xiong et al. 2009; Ren et al. 2010; Du et al. 2011a).

### Pathogen-responsive miRNAs in plants

During the last years, the adoption of deep sequencing technologies has significantly contributed to the discovery of miRNAs in plants. The miRBase registry is the official miRNA repository that keeps the updated annotations on newly discovered miRNAs (http://www.mirbase.org; Kozomara & Griffiths-Jones 2014). Rice is actually the second plant species in number of annotated miRNAs in miRBase, only after *Medicago truncatula*. Based on their nucleotide sequence, miRNAs are grouped into distinct families, each family comprising one or more members. However, the discovery of new miRNAs in plants, including rice, appears to have reached a plateau, and now the challenge is to identify novel miRNAs showing species- or tissue-specific expression and miRNAs that are conditionally expressed (i.e. pathogen-regulated miRNAs).

Although an important number of plant miRNAs have been shown to be regulated by pathogen infection, our understanding of the functional roles of miRNAs in plant disease resistance is far less than that in plant development. For an overview on miRNAs whose accumulation is regulated by different types of pathogen in several plant species, readers are referred to excellent articles (Gupta et al. 2014; Weiberg et al. 2014; Yang and Huang 2014). Functionally characterized miRNAs in plant immunity, mainly in Arabidopsis, are presented in Fig. 1. Among them is miR393, which plays several roles in Arabidopsis defence reactions through regulation of the auxin pathway (miR393) or modulation of PR1 exocytosis (miR393*) (Navarro et al. 2006; Zhang et al. 2011). MiR398 is involved in PTI responses through the control of ROS production, this miRNA targeting two Cu/Zn superoxide dismutase genes (*CSD1* and *CSD2*) and a copper chaperone for superoxide dismutase (Fig. 1). Cu/Zn CSDs are a group of metalloenzymes which act as scavengers of ROS, thus, protecting plants against oxidative stress associated to pathogen infection (Sunkar and Zhu 2004; Jagadeeswaran et al. 2009). Accordingly, transgenic Arabidopsis lines overexpressing miR398 are compromised in resistance to the bacterial pathogen *P. syringae* (Li et al. 2010). In other studies, *Arabidopsis* plants overexpressing either miR400 or miR844 showed much severe disease symptoms compared to wild-type plants when challenged with pathogenic bacteria (*P. syringae* pv tomato DC3000) or fungi (*Botrytis cinerea*) (Park et al. 2014; Lee et al. 2015). MiR400 guides the cleavage of transcripts encoding pentatricopeptide repeat (PPR) proteins, whereas miR844 targets cytidinephosphate diacylglycerol synthase3 (CDS3) transcripts. It is also known that miR160a functions as a positive regulator of PAMP-induced callose deposition, whereas miR398 and miR773 negatively regulate PAMP-induced callose deposition and hence disease resistance to *P. syringae* (Fig. 1).

Various miRNAs are important for ETI responses in different plant species (Fig. 1). A miRNA superfamily comprising miR482 and miR2118 that target NBS-LRR genes was described in tomato (Shivaprasad et al. 2012). Expression of miR482 allows the simultaneous silencing of multiple *R* gene family members from dsRNAs derived from a primary miR482-targeted *R* gene transcript. In *Medicago truncatula*, Zhai et al. (2011) described three abundant miRNA families, miR2109, miR472 and miR1510 that target diverse members of the NB-LRR class of *R* genes and trigger the production of trans-acting siRNAs (ta-siRNAs). In other studies, Li et al. (2012) identified two tobacco miRNAs (nta-miR6019 and nta-miR6020) that guide cleavage of transcripts of the TIR-NB-LRR immune receptor *N* that confers resistance to tobacco mosaic virus (TMV). Co-expression of *N* with nta-miR6019 and nta-miR6020 resulted in attenuation of *N*-mediated resistance to TMV, further supporting that these miRNAs have functional roles in *N* gene regulation. Cleavage of *N* transcripts by nta-miR6019 also triggers the production of secondary siRNAs in phase with the miR6019 cleavage site. Taken together, these findings suggest the existence of a miRNA-mediated regulation of disease resistance genes, and that some of these regulatory miRNAs trigger the production of secondary siRNAs from their *R* gene targets. Boccara et al. (2014) demonstrated that miR472 targets CC-NB-LRR disease resistance genes and act in cooperation with RDR6. They function as negative regulators of PTI and ETI responses to *P. syringae* in Arabidopsis.

An important number of miRNAs that control developmental processes and hormone signalling are also known to be regulated during pathogen infection. In addition to the miR393-mediated regulation of auxin receptors, the expression of several Auxin Response Factors (ARFs) is known to be regulated by different miRNAs in plants (Khraiwesh et al. 2012; Liu et al. 2014) Among them are miR160 (targeting *ARF10*, *ARF16* and *ARF17*) and miR167 (targeting *ARF6* and *ARF8*) (Rhoades et al. 2002). Additionally, *ARF2*, *ARF3* and *ARF4* are indirectly under the control of miR390. Here, miR390 cleaves the non-coding *TAS3* gene for the production of endogenous siRNAs (ta-siRNAs) that target *ARF* transcripts (Allen et al. 2005; Allen et al. 2015). While auxin signalling pathway is regulated by miR160, miR167, miR390 and miR393, the JA biosynthetic pathway is under the control of miR319 and miR159, and miR159 regulate the ABA signalling pathway (Curaba et al. 2014). Altogether, these findings point to an essential role of miRNA-mediated regulatory mechanisms controlling auxin signalling which, in turn, might have an impact in plant disease resistance. Presumably, the ability of a plant to adjust developmental programs and hormone signalling pathways during infection conditions might enhance the plant’s ability to escape from, resist or compensate for disease.

### miRNAs involved in immunity against the rice blast fungus

Great efforts have been made during the last years for the characterization of rice miRNAs accumulating in different tissues and/or developmental stages (e.g. seedlings, shoot, root, panicles), or in response to abiotic stresses (e.g. drought, salt, temperature and nutrient stress) (Jeong et al. 2011; Campo et al. 2013; Li et al. 2013). Also, a substantial fraction of the rice miRNA transcriptome has been reported to be pathogen-responsive (Li et al. 2010; Campo et al. 2013; Baldrich et al. 2015). Unfortunately, the exact role of most of these pathogen-regulated miRNAs in rice immunity remains elusive. Only certain miRNAs have been functionally characterized in the interaction of rice plants with the fungal pathogen *M. oryzae* or during infection with viral pathogens. The contribution of rice miRNAs to antibacterial resistance is less understood, thus reflecting the important gap that occurs in our knowledge of the biological function of rice miRNAs.

The information gained by deep sequencing of small RNA populations revealed dynamic alterations in the expression of an important number of rice miRNAs in response to infection by the rice blast fungus *M. oryzae* or treatment with elicitors prepared from this fungus (Campo et al. 2013; Li et al. 2013; Baldrich et al. 2015). Because miRNAs provide the quantitative regulation of target gene expression, rather than on-off regulations, the observed dynamic responses on miRNA accumulation during pathogen infection would provide the fine-tuning of gene expression in physiological processes contributing to disease resistance. Deep sequencing of the sRNA transcriptomes also allowed the confident identification of previously unknown miRNAs whose expression is regulated by treatment with *M. oryzae* elicitors in rice (Campo et al. 2013; Baldrich et al. 2015). Combined sRNA and degradome analysis revealed the existence of regulatory networks enriched in elicitor-responsive rice miRNAs supported by the identification of their corresponding target genes, such as those associated with hormone signalling and crosstalk between defence-related hormones (ET, SA, JA, auxin) and polyamine biosynthesis. An important number of miRNAs that are regulated by *M. oryzae* elicitors are known to be involved in small RNA pathways, including miRNA and heterochromatic pathways. Among them are miR162 and miR168 (targeting *DCL1* and *AGO1* transcripts, respectively), this observation pointing to a pathogen-regulation of the miRNA machinery itself (Baldrich et al. 2015). Moreover, silencing of *dcl1* has been shown to have a positive impact on resistance to *M. oryzae* infection through the activation of basal defence mechanisms (Zhang et al. 2015). In Arabidopsis, however, a phenotype of susceptibility to infection by *P. syringae* is observed in *dcl1* mutants (Navarro et al. 2008). Thus, differences in the outcome of the interaction in loss-of-function mutants of *dcl1* might be dependent on the host (rice, Arabidopsis) and/or the type of pathogen (fungi, bacteria) .

Rice miRNAs for which a function in blast disease resistance has been demonstrated are miR160a, miR398b and miR7695 (Campo et al. 2013; Li et al. 2013). MiR160 targets Auxin Response Factors (ARFs) involved in auxin signalling. As previously mentioned, auxins have a crucial role in development and control plant immune responses in both Arabidopsis and rice plants (Navarro et al. 2006; Domingo et al. 2009). Overexpression of miR160a or miR398b in transgenic rice results in increased H_2_O_2_ accumulation at the infection site and up-regulation of defence gene expression (i.e. *PR1* and *PR10*) and enhanced resistance to *M. oryzae* (Li et al. 2013).

MiR7695 is a rice-specific miRNA that negatively regulates the accumulation of an alternatively spliced transcript of the *OsNramp* (Natural resistance-associated macrophage protein 6) gene (Campo et al. 2013). NRAMP proteins are involved in the transport of divalent metals and maintenance of metal homeostasis in a wide range of organisms, including plants. Most importantly, overexpression of miR7695 has been shown to confer resistance to infection by the rice blast fungus *M. oryzae* (Campo et al. 2013). The finding that miR7695 positively regulate immunity against the rice blast fungus raises interesting questions concerning the role of metal homeostasis in disease resistance. Additionally, miR7695 represents a recently evolved miRNA that experienced natural and domestication events during rice evolution (Campo et al. 2013).

Understanding the specific function of pathogen-regulated miRNAs and their relevance in disease resistance in rice will require further investigation *in vivo*. The use of transgenic plants with altered expression of pathogen-regulated miRNA or their target genes, and plant lines with attenuated activity of specific miRNAs (e.g. miRNA target mimics, see below) will be key to the future exploration of the contribution of miRNAs to disease resistance.

### MiRNAs in rice/virus interactions

High-throughput sequencing has been also used to monitor alterations in the expression of miRNAs in virally-infected rice plants (Du et al. 2011a; Guo et al. 2012; Sun et al. 2015). Particularly, small RNA profiling of rice plants infected by two distinct viruses, Rice Dwarf Virus (RDV) or Rice Stripe Virus (RSV), demonstrated that infection by one or another virus had distinct impacts on the rice small RNA population (Du et al. 2011a). Some conserved miRNAs respond to RSV but do not respond to RDV, and the other way around. Besides, RSV infection, but not RDV infection, enhanced the accumulation of some rice miRNA* (and not their corresponding miRNAs). MiRNAs that are responsive to infection by the Rice black streaked dwarf virus (RBSDV) or by RSV in rice plants were also described (Guo et al. 2012; Sun et al. 2015). Although some miRNAs exhibited a similar response to RBSDV infection in roots and leaves, many miRNAs had different expression patterns depending on the tissue (Sun et al. 2015). In addition to miRNAs themselves, components of the pathway for miRNA biogenesis and function might also play a role in disease resistance to viral pathogens (Du et al. 2011a). For instance, *dcl2* is known to be up-regulated whereas *dcl3a* and *dcl3b* are down-regulated during RSV infection (Du et al. 2011a). Whether *dcl2* and *dcl3* have an antiviral role in rice still remains to be elucidated. Concerning RDRs, silencing of *rdr6* enhances susceptibility to RSV infection in rice (Jiang et al. 2012). Expression of the rice *AGO18* gene was found to be induced upon infection by RSV and RDV, and transgenic expression of *AGO18* has been shown to confer broad-spectrum virus resistance in rice. The antiviral function of AGO18 depends on its activity to sequester miR168 to alleviate repression of rice AGO1 which is essential for antiviral RNA interference AGO1 (Wu et al. 2015).

### Applications of miRNA-based strategies for rice improvement

The use of miRNA-based technology provides a smart solution for sequence-specific cleavage of any designated target transcript with a great potential for biotechnological approaches for crop protection. Certain miRNAs have proven to be useful for the control of agronomic traits in rice biotechnology (e.g. yield, quality, stress tolerance) (Zhou and Luo 2013; Kamthan et al. 2015; Zheng and Qu 2015). For instance, miR397 overexpression was described to improve rice yield by increasing grain size and promoting panicle branching (Zhang et al. 2013), whereas rice plants overexpressing miR319 had wider leaf blades and enhanced cold tolerance (Yang and Huang 2014). As previously mentioned, disease resistant transgenic rice have been produced by overexpressing either miR160, miR398 or miR7695 (Campo et al. 2013; Li et al. 2013), thus supporting the potential of *MIR* genes to prevent rice disease. A better knowledge of the mechanism governing miRNA function is, however, required to avoid potential undesirable trade-off effects in transgenic plants with altered expression of a miRNA of interest (e.g overexpressor or knock-out transgenic plants).

Artificial microRNAs (amiRNAs) also represents a miRNA-based strategy to target any gene of interest while providing a highly specific approach for gene silencing in plants (Ossowski et al. 2008). The amiRNAs can be created by exchanging the miRNA/miRNA* sequence within a miRNA precursor (transgene) with a sequence designed to match the target gene. Another example that illustrates the usefulness of the miRNA-based technology for plant genetic engineering is the artificial target mimics system. Target mimicry is an endogenous regulatory mechanism that plants use to negatively regulate the activity of specific miRNAs (Franco-Zorrilla et al. 2007). Here, an endogenous long non-coding RNA (*IPS1*, *Induced by Phosphate Starvation1*) binds to miR399 but the pairing is interrupted by a mismatched loop at the expected miRNA cleavage site, which abolishes the cleavage effect (Franco-Zorrilla et al. 2007). In this way, IPS1 serves as a decoy for miR399 and interferes with the binding of miR399 to its target. A genome-wide computational prediction of endogenous miRNA mimics was performed in Arabidopsis and rice in which dozens of target mimics were identified (Meng and Shao 2012) These findings suggest that target mimicry may be widely implicated in regulating miRNA activities *in planta*, including rice. The miRNA target mimicry and artificial miRNA technologies have proven to be useful tools to decipher the function of miRNAs of interest and should have broad applicability for improvement of rice disease resistance. Supporting this notion, a transgenic approach was developed to improve panicle exertion of a rice cytoplasmic male sterile rice line by using a combination of artificial miRNA and artificial target mimic (Chen et al. 2013).

Social concerns raised by the use of genetic modified organisms should be also addressed and continuous scientific efforts are necessary for the safety assessment of transgenic rice. In this respect, new technologies for precise, efficient gene targeting or genome editing such as the TALEN and the CRISPR/Cas9 system have emerged as an alternative to transgenic methods (Miao et al. 2013; Zhang et al. 2014). In particular, the TALEN technology has been successfully used to develop disease-resistant rice plants that do not contain foreign DNA by targeting the rice susceptibility gene *Os11N3* (Li et al. 2012). But still most of the miRNA functions are unknown which means that research efforts need to be directed towards the functional characterization of rice miRNAs playing a role in disease resistance.

## Conclusions

Diseases caused by pathogens, such as fungi, bacteria or viruses, continuously threaten global rice production. Extensive data have shown that an important miRNA repertoire can respond to different types of pathogens in rice. Our understanding of miRNA-mediated processes underlying plant immunity is, however, far from complete. Besides regulating gene expression critical for plant growth and development, miRNAs can also play a fundamental role in disease resistance in plants. Further investigation on miRNA-mediated regulated processes in rice-pathogen interactions will have important implications in designing novel strategies for disease control which, in turn, might contribute to improve rice productivity. MiRNA will be also useful as biomarkers for disease resistance traits in breeding programs.
